# Integrated analysis of single-cell and bulk transcriptomes reveals the prognostic value of polyamine metabolism biomarkers and immune microenvironment features in gastric cancer

**DOI:** 10.3389/fimmu.2025.1658975

**Published:** 2026-01-30

**Authors:** Kailun Chen, Yuteng Chen, Qinqin Hu, Jie Zheng, Yanan Liu

**Affiliations:** 1 Department of General Internal Medicine (Gastroenterology and Respiratory Medicine), Zhejiang Provincial People’s Hospital (Affiliated People’s Hospital), Hangzhou Medical College, Hangzhou, Zhejiang, China; 2 Department of Endocrinology and Metabolism, Shaoxing No.2 Hospital Medical, Shaoxing, Zhejiang, China; 3 Department of General Internal Medicine, Tongde Hospital of Zhejiang Province, Hangzhou, Zhejiang, China; 4 Department of Teaching, Tongde Hospital of Zhejiang Province, Hangzhou, Zhejiang, China; 5 Department of Oncology, Weihai Municipal Hospital, Cheeloo College of Medicine, Shandong University, Weihai, Shandong, China

**Keywords:** gastric cancer, biomarker, single cell, machine learning, polyamine metabolism

## Abstract

**Background:**

Gastric cancer (GC) remains a lethal malignancy with limited prognostic biomarkers. Dysregulated polyamine metabolism promotes tumor progression and immune evasion, yet its clinical implications in GC are poorly characterized.

**Methods:**

We conducted an integrative analysis using bulk RNA-seq and single-cell RNA-seq data to investigate the prognostic significance of polyamine metabolism-related genes (PMRGs) in GC. A total of 59 PMRGs were curated and used to score cells via AUCell. High- and low-scoring cells were subjected to differential gene expression, enrichment, and pseudotime trajectory analyses. Prognostic modeling was performed using 10 machine learning algorithms across multiple combinations, followed by validation and nomogram construction. Immune infiltration, immune checkpoint expression, cell-cell communication, and immunotherapy response were evaluated. Drug sensitivity and tumor mutational burden (TMB) were analyzed using public pharmacogenomic datasets.

**Results:**

Single-cell analysis identified PMRGs-driven heterogeneity across 11 cell types, with fibroblasts and macrophages showing enhanced signaling in high-risk populations. A 13-gene signature was constructed using StepCox and elastic net, achieving robust prognostic performance (Train dataset AUCs: 0.67-0.70; Validation dataset AUCs: 0.64-0.67). High-risk patients exhibited enriched stromal-immune interactions, elevated immune infiltration, higher Tumor Immune Dysfunction and Exclusion (TIDE) scores, and poorer immunotherapy response. Low-risk patients had higher TMB and sensitivity to 5-Fluorouracil, Docetaxel, Doxorubicin and Paclitaxel.

**Conclusion:**

Polyamine metabolism shapes both cellular heterogeneity and the immune microenvironment in gastric cancer. Our integrated model may provide potential guidance for prognostic stratification and therapeutic decision-making in clinical oncology.

## Highlights

- Developed a robust 13-gene prognostic model based on polyamine metabolism using multi-algorithmic machine learning.
- Integrated single-cell and bulk transcriptomic data to uncover immune and metabolic heterogeneity in GC.
- Signature genes correlated with sensitivity to standard chemotherapeutics, informing personalized treatment strategies.

## Introduction

Gastric cancer (GC) continues to pose a significant global health burden, currently ranking among the top five most frequently diagnosed cancers and standing as the third most common cause of cancer mortality worldwide (1). Histopathological analysis indicates that over 95% of GC cases are adenocarcinomas in origin (2). The high mortality rate associated with GC is largely attributed to substantial molecular heterogeneity, delayed diagnosis, and limited treatment options for advanced-stage disease (3, 4). Although advances in genomic stratification have been made, such as the classification of four molecular subtypes proposed by The Cancer Genome Atlas (TCGA) (5), the clinical translation of these molecular insights has been limited. This highlights the need for integrative biomarkers that capture both molecular alterations and dynamic tumor microenvironment (TME) features (6).

Among cancer-associated metabolic pathways, polyamine metabolism plays a pivotal role in regulating cell proliferation, differentiation, and stress adaptation (7, 8). Under physiological conditions, polyamines such as putrescine, spermidine, and spermine contribute to cellular homeostasis by stabilizing nucleic acid structures and regulating translational processes (9). Dysregulated polyamine metabolism, however, fosters malignant transformation, invasion, and therapeutic resistance (10). Enzymes such as adenosylmethionine decarboxylase 1 (AMD1) are frequently upregulated in GC and linked to tumor aggressiveness (11). Moreover, Helicobacter pylori infection can remodel polyamine metabolism via activation of ARG2, ODC, and SMO, thereby promoting inflammation, DNA damage, and immune escape (12, 13). These findings underscore the importance of polyamine metabolism in gastric tumorigenesis. Nevertheless, previous studies have mainly focused on bulk-tissue expression or metabolic enzyme alterations, leaving the cellular and microenvironmental context of polyamine metabolism in GC insufficiently characterized.

Recent advances in single-cell RNA sequencing (scRNA-seq) have provided high-resolution maps of the gastric tumor microenvironment, uncovering distinct cellular states and intercellular interactions that influence tumor progression and therapy response (14).

Integrating single-cell datasets with bulk transcriptomics and applying machine-learning frameworks enables robust biomarker discovery and improves clinical generalizability (15). Building on these developments, our study integrates single-cell and bulk transcriptomic data with a multi-algorithm machine-learning pipeline to investigate polyamine metabolism in gastric cancer. We delineate cell type-specific polyamine activity, construct and externally validate a 13-gene prognostic signature, and link it to immune landscapes, genomic alterations, and predicted therapeutic vulnerabilities, thus extending prior bulk- or enzyme-focused studies by resolving the cellular context and translational relevance of polyamine metabolism.

## Materials and methods

### Data acquisition

mTranscriptomic profiles (32 normal and 375 tumor samples), along with somatic mutation and clinical annotation data for stomach adenocarcinoma (STAD), were obtained from TCGA repository. To reduce potential bias from non–cancer-related deaths or perioperative complications, patients with an overall survival of less than 30 days were excluded, resulting in 335 eligible cases for downstream analyses. The Gene Expression Omnibus (GEO) dataset GSE26901 (n=109) served as an external validation cohort. Single-cell transcriptomic profiles were retrieved from the GSE183904 dataset. A total of 59 genes associated with polyamine metabolism (PMRGs) were selected based on the REACTOME_METABOLISM_OF_POLYAMINES pathway defined in the MSigDB database.

### Single-cell transcriptome analysis

The GSE183904 dataset was analyzed using the Seurat package, with the top 2,000 highly variable genes selected through the application of the *FindVariableFeatures()* function. Data integration and batch correction were performed using *Harmony* R package on the top 20 principal components with default parameters. UMAP reduction was applied using Harmony-corrected embeddings with dims = 1:20 and default settings. Cells with >250 genes, <20% mitochondrial content, and ≥5 reads per gene were retained, yielding 73,846 cells. Data were normalized and scaled; PCA was performed followed by clustering at resolution 0.3. Annotation was manually performed using canonical cell markers. *Scissor* R package identified cells associated with high- and low-risk patients by integrating single-cell expression profiles, bulk TCGA expression, and risk-group labels. *AUCell* was employed to quantify the activity of PMRGs at the single-cell level, and cells were subsequently stratified into high- and low-score groups according to the median module activity. Differential PMRGs expression was assessed using *FindAllMarkers()*. For CellChat analysis, interactions were computed using *computeCommunProb()* with min.cells = 10, and interactions with a communication probability below 0.05 were filtered out. Single-cell pseudotime trajectories were inferred via *Monocle2* to explore differentiation dynamics.

### Prognostic model construction via machine learning

A total of 408 PMRGs-derived differentially expressed genes (PMRDEGs) were subjected to prognostic feature selection across 10 machine learning methods and 101 model combinations, including Support Vector Machine (SVM), Least Absolute Shrinkage and Selection Operator (LASSO), Gradient Boosting Machine (GBM), Random Forest (RF), Elastic Net, Stepwise Cox Proportional Hazards Regression, Ridge Regression, CoxBoost Algorithm, Supervised Principal Components (SuperPC), and Partial Least Squares Regression for Cox Model (plsRcox). Model development was implemented using the *ML.Dev.Prog.Sig()* function from the *Mime1* R package. The Cancer Genome Atlas - Stomach Adenocarcinoma (TCGA-STAD) cohort served as the training dataset, while GSE26901 was used for external validation. Internal validation within the training set was performed via 10-fold cross-validation to evaluate model stability and prevent overfitting. The random seed was set to 5201314 to ensure reproducibility. Candidate prognostic genes were first screened by univariate Cox regression (p < 0.05), and model selection was based on the highest Harrell’s concordance index (C-index) across all algorithmic combinations. Based on the median risk score, patients were stratified into high- and low-risk subgroups. Kaplan-Meier survival analysis was conducted to assess prognostic differences, while the model’s predictive accuracy was validated through calibration plots and time-dependent receiver operating characteristic (ROC) curve analysis.

### Construction and evaluation of a prognostic nomogram

To determine the independent prognostic significance of the risk score, both univariate and multivariate Cox regression models were applied, integrating clinical features. A nomogram predicting 3- and 5-year overall survival probabilities was developed using the *rms* R package. Its performance was assessed through calibration curve consistency and decision curve analysis (DCA) to evaluate clinical utility.

### Functional enrichment analysis

Genes differentially expressed between high- and low-risk cohorts (threshold: |log_2_FC| > 1, adjusted p < 0.05) were identified via the *limma* package. Gene Set Enrichment Analysis (GSEA) was subsequently conducted using both the standalone GSEA tool (v4.3.3) and the *clusterProfiler* R package to explore enriched biological processes (GO) and pathways (KEGG).

### Immune infiltration analysis

Immune infiltration was evaluated using ssGSEA and CIBERSORT algorithms. Immune checkpoint gene expression, Immunophenoscore (IPS) scores from The Cancer Immunome Atlas (TCIA), and Tumor Immune Dysfunction and Exclusion (TIDE) scores were compared between risk groups. Immunotherapy response was predicted in the IMvigor210 anti-PD-L1 cohort by categorizing responders (R) and nonresponders (NR) relative to risk scores.

### Tumor mutational burden and drug sensitivity prediction

STAD mutation data from TCGA were used to calculate tumor mutational burden (TMB), comparing high- and low-risk groups via Wilcoxon test. The top 20 mutated genes were visualized using *GenVisR*.

For drug sensitivity analysis, gene-drug interaction data were obtained from the DGIdb database. Correlations between model gene expression and compound activity (IC50) were evaluated using *CellMiner*, which integrates NCI-60 pharmacogenomic profiles. Gene expression and drug sensitivity data were log-transformed, normalized, and Spearman correlations were computed. To further predict chemotherapeutic responses, the pRRophetic R package was employed to estimate drug IC50 values. The model utilized ridge regression trained on the GDSC pharmacogenomic dataset, assuming consistent gene-drug associations between GDSC and TCGA-STAD transcriptomic profiles. Batch effects were corrected using the “Empirical Bayes” method, and the average expression of duplicated genes was computed using the *avereps()* function. Patients were stratified into high- and low-risk groups to compare predicted IC50 values.

### Cell culture and RT-qPCR

This study employed normal gastric epithelial cells (GES-1) and human gastric cancer cells (AGS) for experimentation. Under strict aseptic conditions, both cell lines were cultured in RPMI-1640 medium supplemented with 10% fetal bovine serum. Total RNA was extracted from the cells using TRIzol reagent, and cDNA was synthesized via reverse transcription with PrimeScript™ RT Master Mix. RNA and cDNA concentrations were quantified using a NanoDrop 2000 spectrophotometer. RT-qPCR was performed on a QuantStudio 5 real-time fluorescence quantitative PCR system, with primer sequences detailed in **Table 1**. GAPDH served as the internal reference gene, and relative expression levels were calculated using the 2^(-ΔΔCT) method.

**Table 1:** Primer sequences for RT-qRCR.

Gene	Forward primer	Reverse primer
ANXA5	TGGCTCAAGCCTGGAAGATG	GCATCAGGGTCTCTGTTAGCC
ARGLU1	AAGCACAACAAGAAGCGCAG	GAGATTTGGAACGCTTCCGC
CD59	GCGCCGCCAGGTTCT	GCTCATTTTCCCTCAAGCGG
CDK5RAP3	CTTTGGGACGTCTCACCGAC	AGCTTGCTGGTCTGGATGTC
CXCR4	AGCGTCTCAGTGCCCTTTTG	GGTAGCGGTCCAGACTGATG
GLA	TCCTGCATCAGTGAGAAGCTC	CAGGCCACTCCTTTACCCAG
PER1	GCAGGCCAACCAGGAATACT	ACAGAAGCGGATAGGGGAGT
RGS2	ATTCAGCCTGGGTGTTCAGG	AGACACCACGTTCAGACCAC
TAP1	TACTGCTACTTCTCGCCGAC	ACTGACAACGAAGGCGGTAG
TCIRG1	CTGCCTACACCTGCGTGAGT	CCCTCACTCACTTGACCCTC
ZFP36	CACCTCTTCCCTGCCCAAAT	ACCAGGAGACACTGGAACCT
GPADH	CTGGGCTACACTGAGCACC	AAGTGGTCGTTGAGGGCAATG

### Statistical analysis

All statistical analyses were conducted in R (v.4.1.0) unless otherwise noted. P-value < 0.05 was considered significant. Survival curves were compared via log-rank test, and other comparisons used Wilcoxon or appropriate tests.

## Results

### Single-cell landscape of polyamine metabolism in gastric cancer

**Figure 1:**
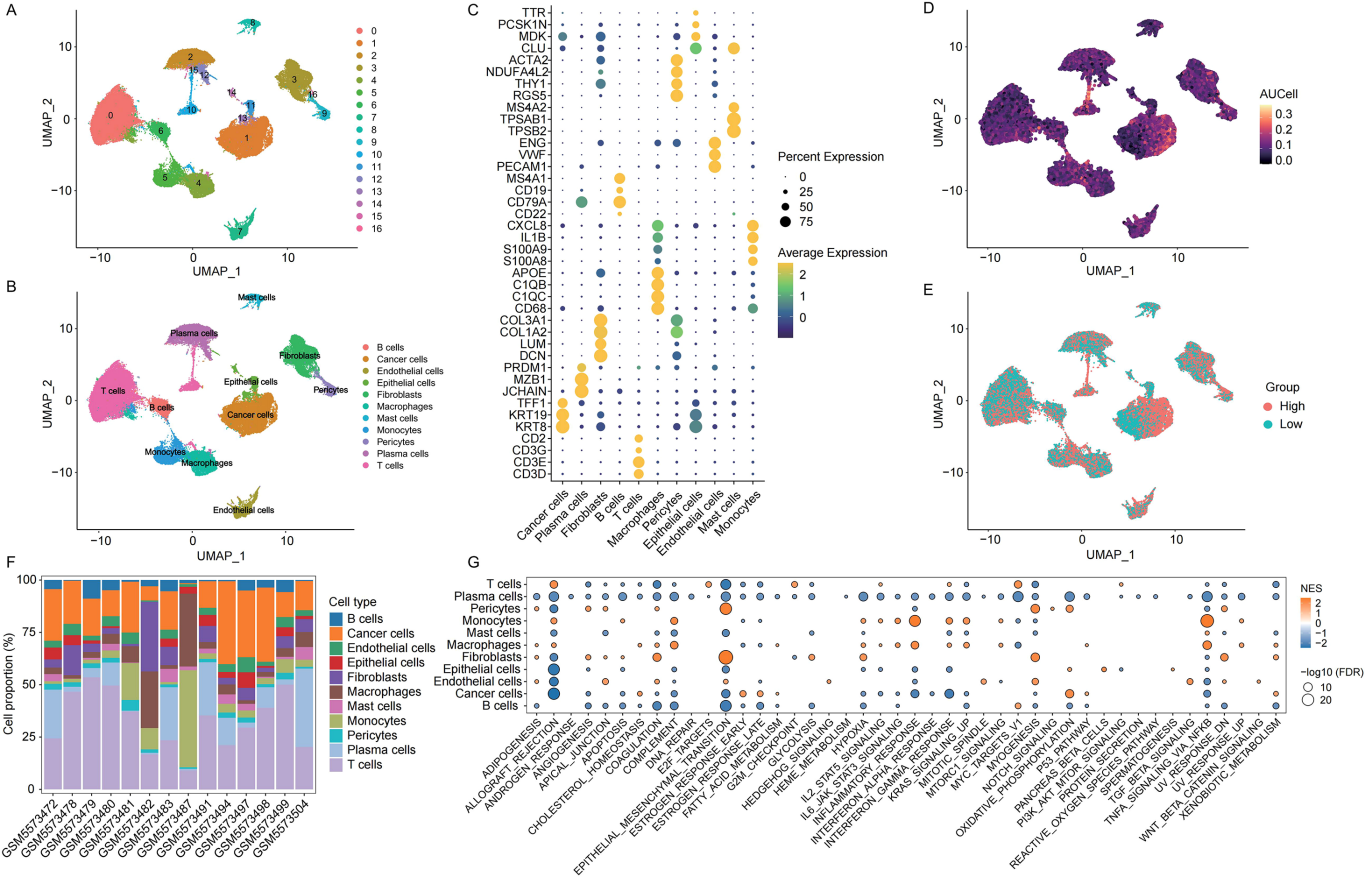
Single-cell transcriptomic analysis of gastric cancer and characterization of polyamine metabolism. **(A)** UMAP plot illustrating the clustering of cells from gastric cancer single-cell RNA sequencing data. **(B)** UMAP visualization of annotated cell subtypes. **(C)** Bubble plot showing representative marker genes for each identified cell type. **(D)** UMAP distribution of AUCell scores for polyamine metabolism-related gene sets. **(E)** UMAP plot of cells categorized into high and low AUCell score groups based on the median score. **(F)** Proportional composition of each cell type across individual samples. **(G)** Hallmark pathway enrichment results based on differentially expressed genes across cell clusters.

A total of 73,846 cells were obtained from GC patients in the GSE183904 dataset. Following batch effect removal, normalization, dimensionality reduction, and clustering, cells were annotated into 11 distinct types: T cells, B cells, endothelial cells, epithelial cells, fibroblasts, macrophages, mast cells, monocytes, pericytes, plasma cells, and cancer cells (**Figures 1A, B**; **Supplementary Figure 1**). Representative marker genes for each cell type were visualized using a bubble plot (**Figure 1C**). To further validate the accuracy of cell-type annotations, UMAP plots showing the expression of representative marker genes were generated for each cell population and provided in **Supplementary Figure 2**. Using a curated set of 59 polyamine metabolism-related genes, AUCell scoring stratified cells into high- and low-scoring groups (**Figures 1D, E**). Differentially expressed genes between these two groups were identified as PMRDEGs.

Cell-type composition was assessed across individual samples (**Figure 1F**), followed by functional enrichment analyses of differentially expressed genes (DEGs) identified within each cell population. GO and KEGG pathway analyses indicated that DEGs from T cells were predominantly enriched in T cell-associated signaling cascades—such as the T cell receptor signaling pathway—providing additional support for the accuracy of our cell-type annotations (**Supplementary Figure 3**). Hallmark analysis revealed enrichment of epithelial-mesenchymal transition signatures in fibroblasts and pericytes, while monocytes and macrophages showed enrichment in inflammatory response and TNFα signaling via NF-κB pathways (**Figure 1G**).

### Machine learning-derived prognostic signature

**Figure 2:**
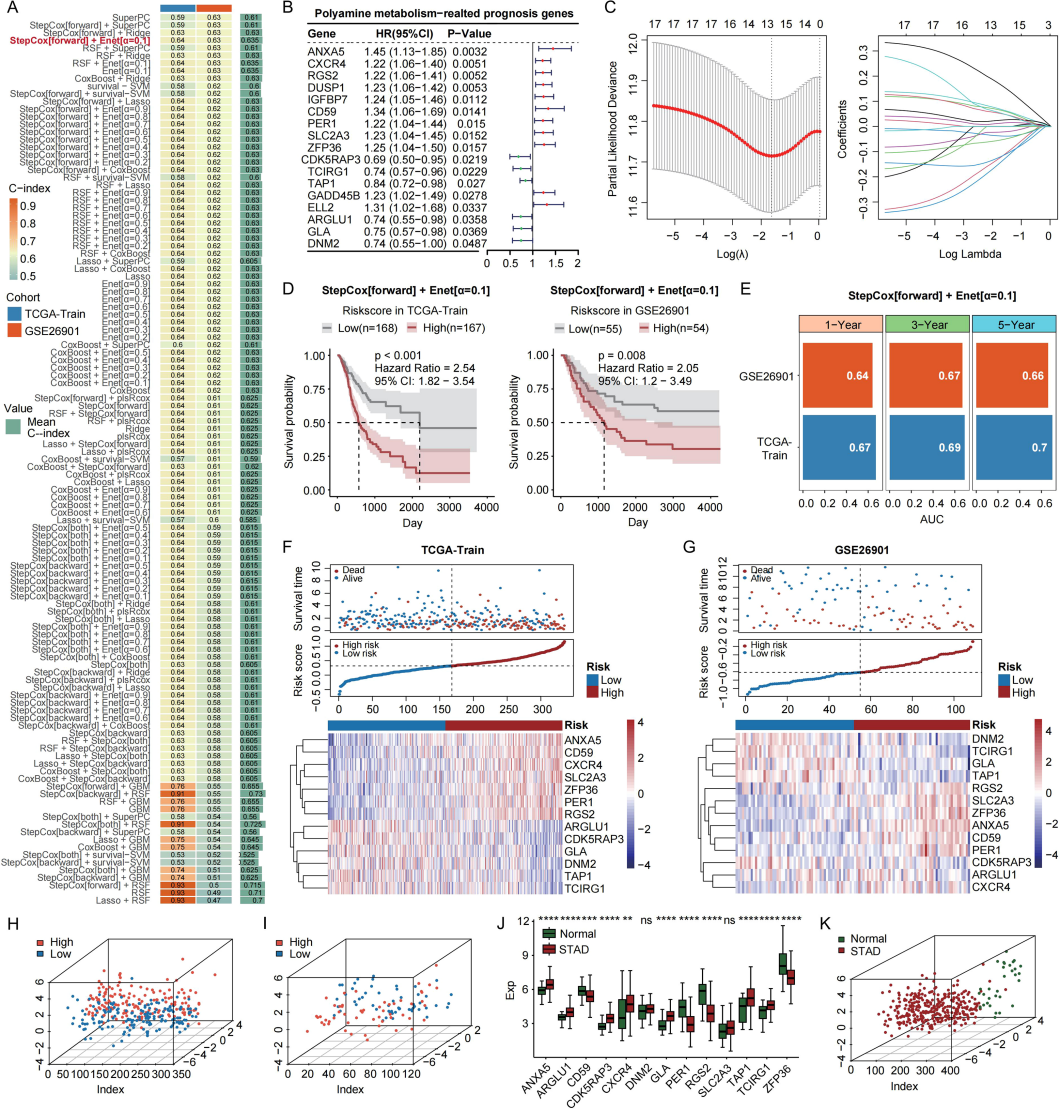
Construction and validation of the prognostic model based on machine learning and gene expression profiles. **(A)** C-index values calculated using multiple combinations of machine learning algorithms. **(B)** Forest plot of univariate Cox regression analysis. **(C)** Coefficient trajectories and cross-validation plot generated from the elastic net model (α = 0.1) across a logarithmic sequence of λ values. **(D)** Kaplan-Meier survival curves for the TCGA training and GEO validation cohorts. **(E)** Time-dependent AUC values at 1, 3, and 5 years in the TCGA training and GEO validation cohorts. **(F)** Distribution of risk scores, survival outcomes, and expression patterns of signature genes across high- and low-risk subgroups within the TCGA cohort and **(G)** independent GEO validation set. **(H)** PCA plot based on selected signature genes effectively delineates high- and low-risk subgroups in the TCGA discovery cohort and **(I)** the GEO validation cohort. **(J)** Profound dysregulation of signature genes observed between tumor specimens and matched normal tissues. **(K)** Detection of mRNA expression levels for 11 genes in different cell lines using RT-qPCR experiments. ns, not significant, *p<0.05, **p<0.01, ***p<0.001.

To construct a prognostic model, we evaluated the expression profiles of 408 PMRDEGs using 10 machine learning algorithms, including CoxBoost, Enet, GBM, Lasso, plsRcox, Ridge, RSF, stepwise Cox, SuperPC, and survival-SVM, as implemented in the *Mime1* R package. A 10-fold cross-validation framework was used within the TCGA training cohort to assess model robustness and minimize overfitting, and the best-performing model was selected based on the highest C-index. (**Figure 2A**). Univariate Cox regression identified 17 prognostically relevant PMRDEGs (**Figure 2B**), which were further optimized using a combination of stepwise Cox (forward) selection and the Enet model (α = 0.1). 13 genes (ANXA5, CD59, CXCR4, SLC2A3, ZFP36, PER1, RGS2, ARGLU1, CDK5RAP3, GLA, DNM2, TAP1, TCIRG1) were ultimately selected to construct the final model (**Figure 2C**). Survival analysis revealed that high-risk patients exhibited significantly worse outcomes in both cohorts (**Figure 2D**). The time-dependent AUCs for 1-, 3-, and 5-year survival were 0.67, 0.69, and 0.70 in TCGA, and 0.64, 0.67, and 0.66 in GSE26901, respectively (**Figure 2E**). Risk score distribution, survival status, and heatmaps of gene expression levels consistently distinguished high- from low-risk groups (**Figures 2F, G**). PCA confirmed clear separation between the two risk groups (**Figures 2H, I**). Additionally, expression levels of the 11 genes significantly differed between tumor and normal tissues, demonstrating their diagnostic potential (**Figure 2J**), RT-qPCR experiments further confirmed these analytical results (**Figure 2K**). To further validate the clinical utility of our model, we compared its predictive performance with ten previously published gastric cancer prognostic models. As shown in **Supplementary Figure 4**, our model achieved higher time-dependent AUC values at 1-, 3-, and 5-year survival, demonstrating superior prognostic accuracy and robustness.

### Clinical applicability and independent prognostic value

**Figure 3:**
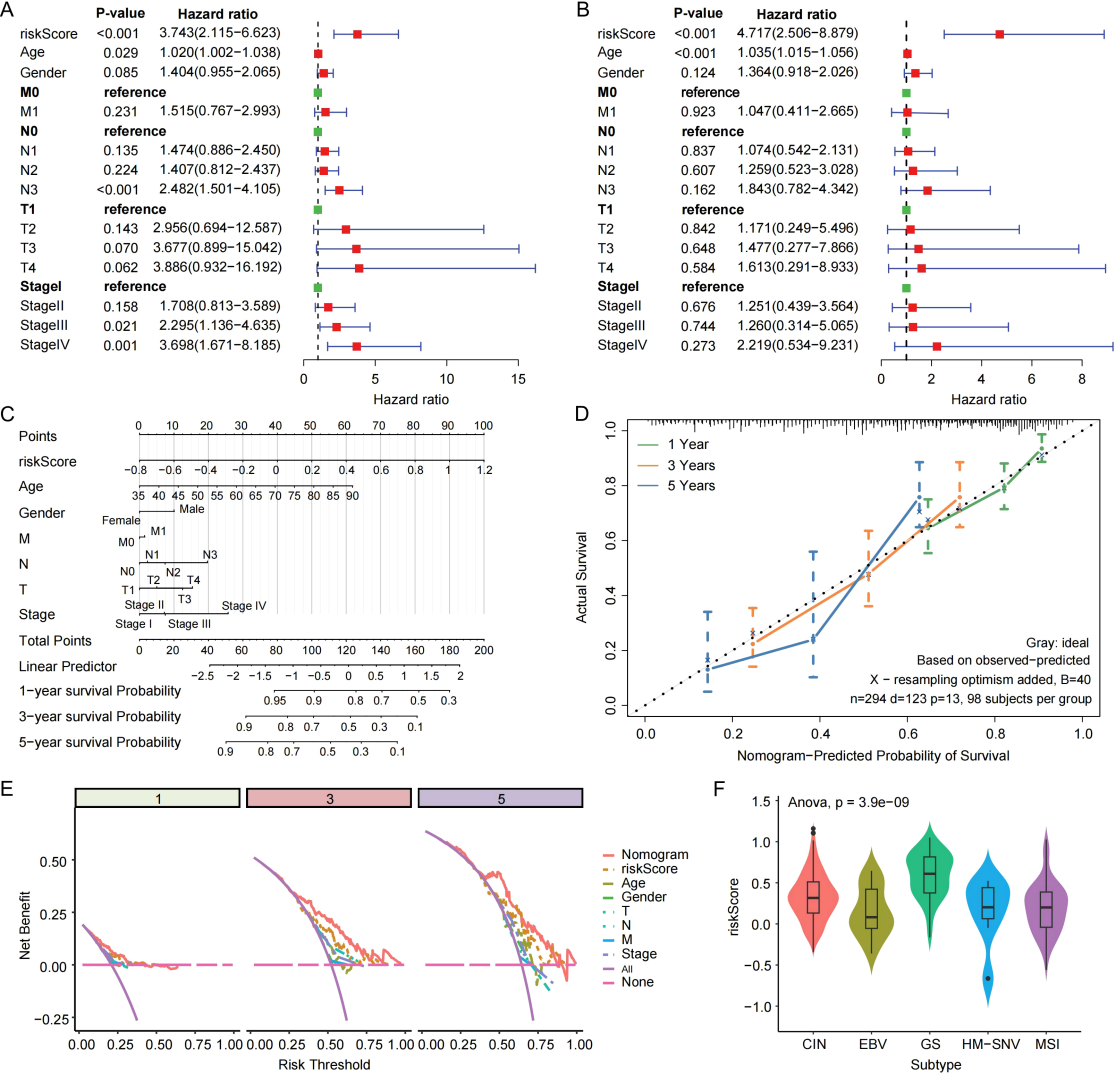
Prognostic value and clinical applicability of the risk model in gastric cancer. **(A)** Univariate Cox analysis identified preliminary prognostic associations visualized via forest plot. **(B)** Multivariate-adjusted forest plot delineated independent predictors of clinical outcomes. **(C)** Integrative nomogram incorporating risk signatures and clinicopathological variables for personalized survival estimation. **(D)** Calibration curves evaluating the agreement between predicted and observed survival at 1, 3, and 5 years. **(E)** Decision curve analysis (DCA) curves for assessing clinical utility at 1, 3, and 5 years. **(F)** Comparison of risk scores across different molecular subtypes of gastric cancer.

Univariate and multivariate Cox regression analyses indicated that both risk score and age were independent prognostic factors in gastric cancer (**Figures 3A, B**). A nomogram integrating clinical features and risk score was constructed to predict individual survival probabilities (**Figure 3C**). Calibration curves across 1-, 3-, and 5-year temporal points revealed superb alignment of nomogram-predicted probabilities with actual observations (**Figure 3D**). Decision curve and clinical impact curve analyses consistently demonstrated favorable net benefit and predictive utility across a range of threshold probabilities (**Figure 3E**; **Supplementary Figure 5**). Furthermore, subgroup survival analyses showed that the risk model retained significant prognostic value in stage I–III patients but not in stage IV, suggesting that its predictive power is more pronounced in earlier disease stages (**Supplementary Figure 6**). Notably, patients with the genomically stable (GS) subtype exhibited significantly higher risk scores (**Figure 3F**). Single-cell analysis revealed that GS subtype patients had a higher proportion of cancer and mast cells, consistent with their poor prognosis (**Supplementary Figure 7**).

### Cell-cell communication networks driven by risk-associated populations

**Figure 4:**
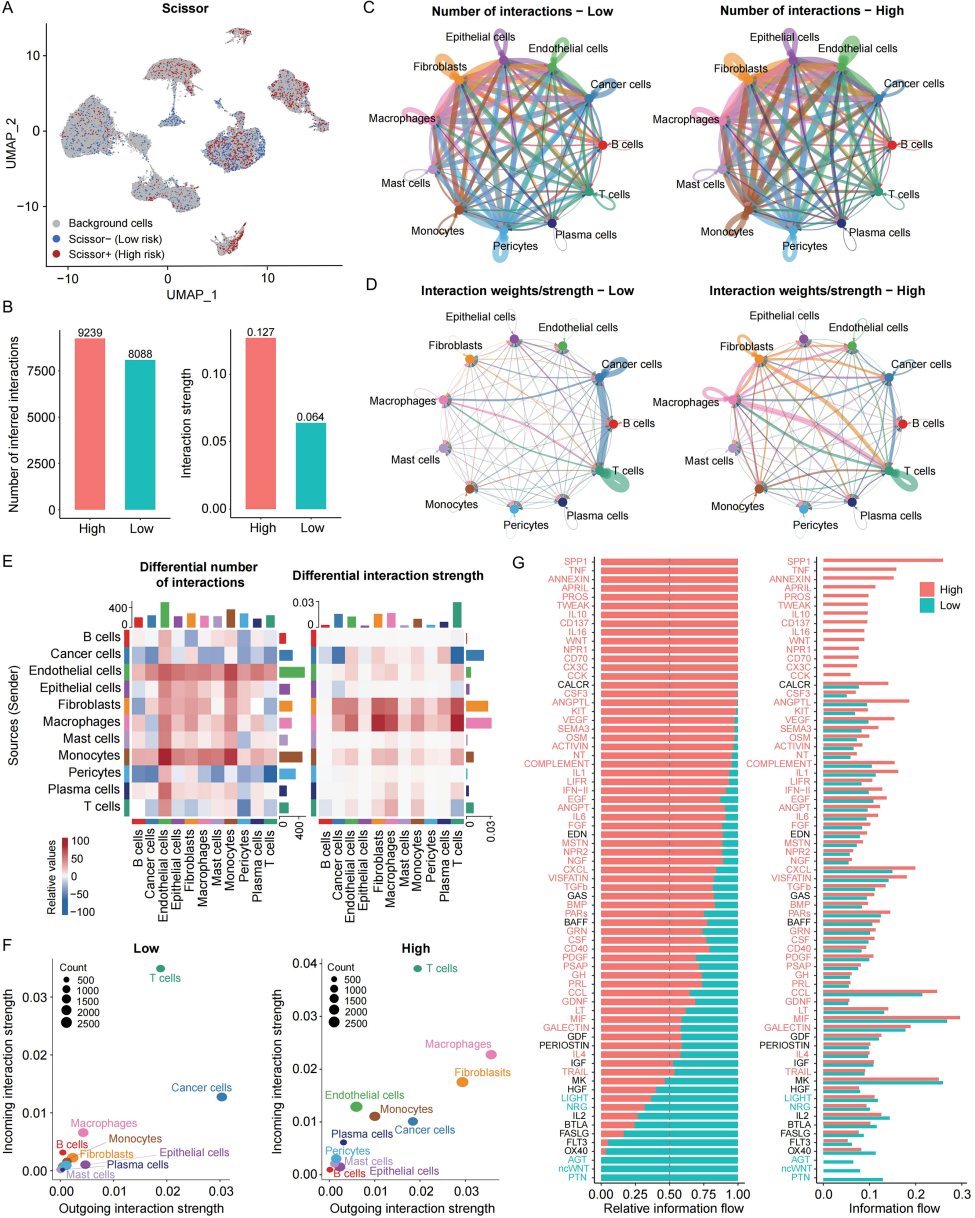
Cell-cell communication patterns between high- and low-risk associated cellular populations. **(A)** UMAP plot depicting cell populations associated with high- and low-risk groups. **(B)** Bar chart comparing the number and strength of intercellular interactions between high- and low-risk associated cell populations. **(C)** Circle plot visualizing the number of interactions and **(D)** strength among cell populations associated with different risk groups. **(E)** Heatmap representing the number and strength of intercellular interactions among risk-related cell populations. **(F)** Incoming and outgoing signaling strengths of individual cells within high- and low-risk associated cell groups. **(G)** Comparative analysis of global signaling pathway activity between high- and low-risk associated cells; red indicates pathways enriched in high-risk related cells, blue denotes enrichment in low-risk related cells, and black represents pathways with no significant difference between groups.

SCISSOR analysis was used to associate specific cell populations with patient risk stratification (**Figure 4A**). CellChat analysis showed that high-risk cells exhibited a greater number and intensity of intercellular interactions compared to low-risk cells (**Figures 4B–D**; **Supplementary Figures 8**, **9**). Interaction heatmaps emonstrated that fibroblasts and macrophages in the high-risk group had significantly enhanced signaling activity (**Figure 4E**). Outgoing signals were primarily driven by cancer cells in the low-risk group and by fibroblasts and macrophages in the high-risk group, whereas T cells exhibited strong incoming signals in both groups (**Figure 4F**). Signaling pathway comparison revealed that high-risk groups were enriched in SPP1, TNF, and ANNEXIN pathways, while low-risk groups showed enrichment in PTN, ncWNT, and AGT pathways (**Figure 4G**). Key ligand-receptor interactions were further statistically validated to confirm their robustness (**Supplementary Tables 1, 2**).

### Functional enrichment of risk-associated transcriptomes

**Figure 5:**
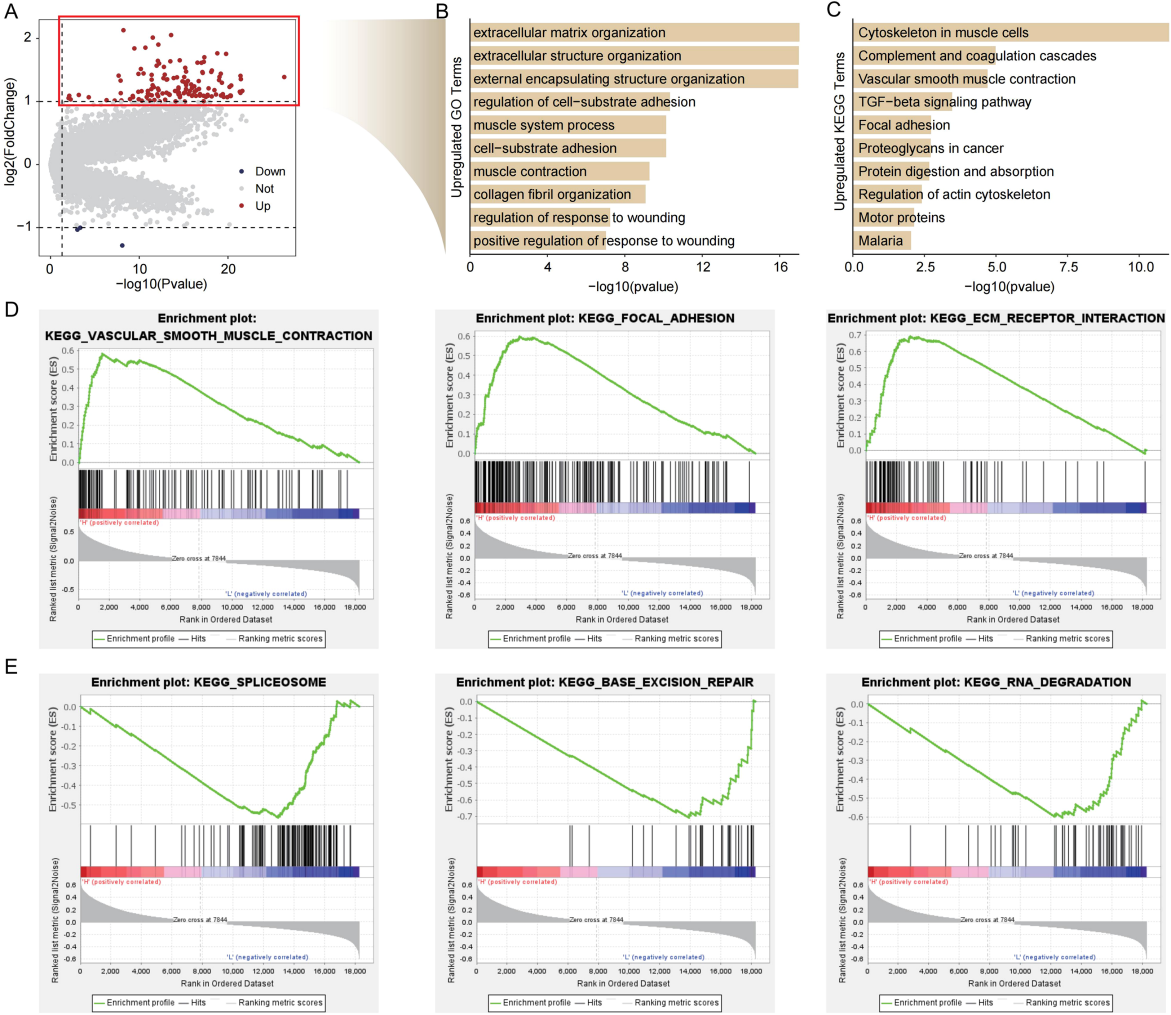
Functional annotation of risk-stratified transcriptional profiles. **(A)** Volcano plot delineating differentially expressed genes (DEGs) between prognostic subgroups. **(B)** Gene Ontology (GO) and **(C)** Kyoto Encyclopedia of Genes and Genomes (KEGG) pathway enrichment of upregulated transcripts in high-risk cohorts. **(D)** Gene Set Enrichment Analysis revealing pathway dysregulation in **(D)** high-risk versus **(E)** low-risk subgroups.

Transcriptomic profiling identified 139 genes with significant upregulation in the high-risk cohort (|log_2_FC| > 1, adjusted p < 0.05), indicative of an aggressive molecular phenotype (**Figure 5A**). GO enrichment highlighted terms related to extracellular matrix organization, while KEGG pathways included TGF-beta signaling, focal adhesion, and proteoglycans in cancer (**Figures 5B, C**). GSEA further revealed that high-risk groups were enriched in vascular smooth muscle contraction and ECM receptor interaction, whereas low-risk groups showed enrichment in spliceosome, base excision repair, and RNA degradation pathways (**Figures 5D, E**). These results demonstrate that high-risk tumors are characterized by extracellular matrix remodeling and stromal activation, suggesting dysregulated polyamine metabolism drives tumor-promoting microenvironments. Conversely, low-risk tumors exhibit enhanced nucleic acid maintenance mechanisms, indicating preserved genomic integrity.

### Immune microenvironment and therapy response stratification

**Figure 6:**
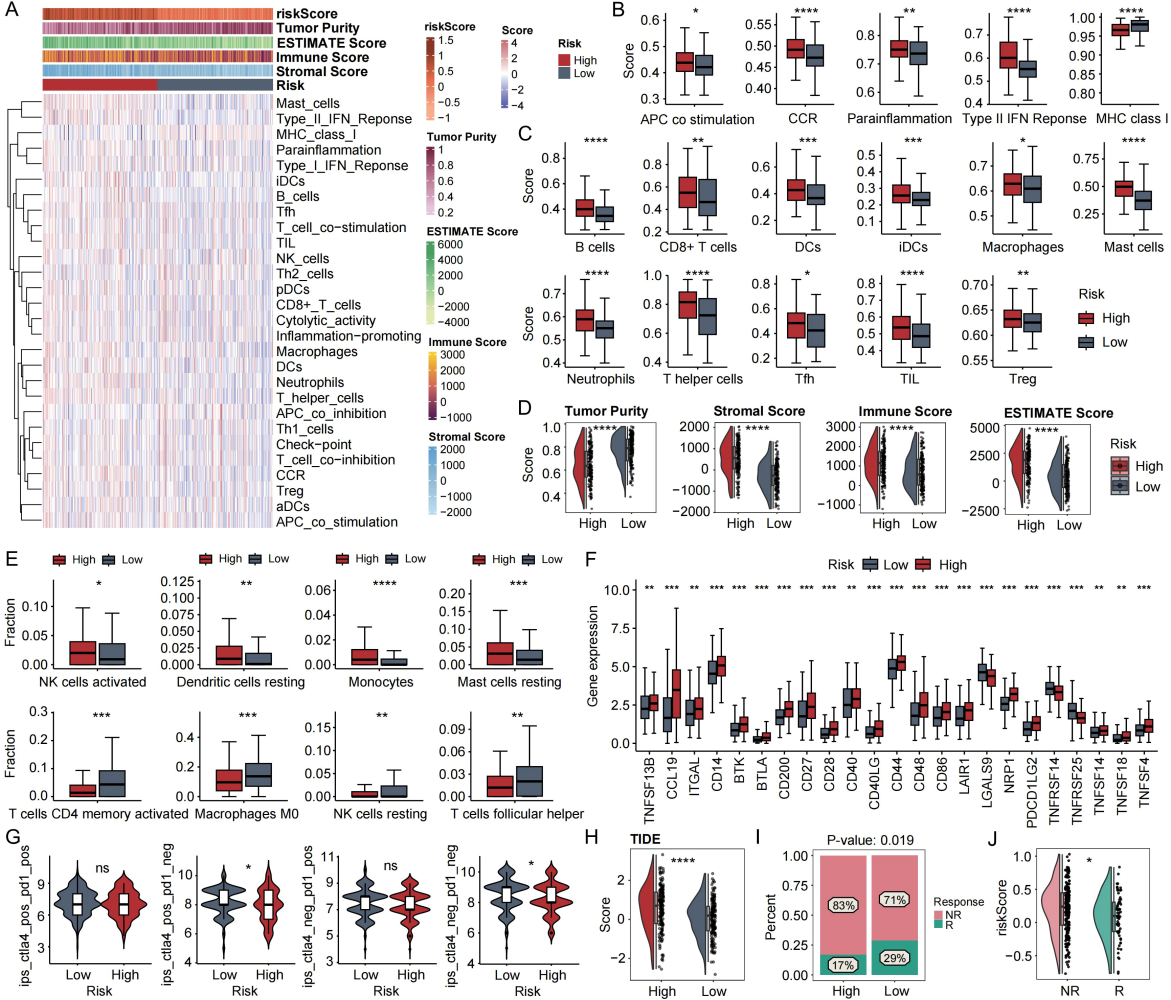
Immune landscape and immunotherapy response prediction based on risk stratification. **(A)** Heatmap illustrating immune infiltration disparities across prognostic risk strata based on ssGSEA. **(B)** Functional immunome divergence and **(C)** cellular composition bias in dichotomized risk cohorts. **(D)** Tumor microenvironmental contrast by ESTIMATE scoring in risk-stratified cohorts. **(E)** Boxplots showing significantly differentially distributed immune cell types between the two risk groups. **(F)** Boxplots of immune checkpoint expression differences between high- and low-risk groups. **(G)** Violin plots displaying differences in Immunophenoscore (IPS) between high- and low-risk groups. **(H)** Violin plots showing TIDE score differences between high- and low-risk groups. **(I)** Stratification of anti-PD-L1 therapeutic outcomes by risk categories in the IMvigor210 cohort. **(J)** Comparison of risk scores between non-responders (NR) and responders (R) in the IMvigor210 cohort. ns, not significant, *p<0.05, **p<0.01, ***p<0.001, ****p-value < 0.0001.

ssGSEA quantification demonstrated augmented immune effector activity and intensified leukocyte infiltration within high-risk cohorts (**Figure 6A**). Specifically, the high-risk stratum exhibited markedly elevated enrichment scores for B lymphocytes, cytotoxic CD8+ T cells, dendritic cells, macrophages, mast cells, neutrophils, and regulatory T cells (Tregs), whereas major histocompatibility complex class I (MHC-I) molecules showed preferential expression in low-risk counterparts (**Figures 6B, C**). ESTIMATE computational deconvolution revealed augmented stromal/immune compartment abundance and composite microenvironment scores, concurrent with diminished tumor purity in high-risk cohorts (**Figure 6D**). CIBERSORT analysis indicated that activated NK cells, resting dendritic cells, monocytes, and resting mast cells were more prevalent in the high-risk group, whereas activated CD4+ memory T cells, M0 macrophages, resting NK cells, and follicular helper T cells were higher in the low-risk group (**Figure 6E**). Dysregulated overexpression of immune checkpoint molecules predominated in high-risk cohorts (**Figure 6F**). Tumor IPS quantification from TCIA demonstrated significantly enhanced scores in low-risk patients, correlating with improved immunotherapy responsiveness (**Figure 6G**). Complementing these findings, TIDE algorithms revealed substantially elevated immune evasion potential in high-risk versus low-risk strata (**Figure 6H**). Building upon immune infiltration profiling, we assessed anti-PD-L1 therapeutic efficacy in the IMvigor210 cohort (n=348). Treatment responses were categorized into four clinically defined groups: progressive disease (PD), stable disease (SD), partial response (PR), and complete response (CR). For analysis, SD and PD were grouped as NR, while CR and PR were grouped as R. The proportion of responders was significantly higher in the low-risk group, and responder patients exhibited significantly lower risk scores compared to non-responders (**Figures 6I, J**).

### Pseudotime analysis reveals T cell differentiation dynamics

**Figure 7:**
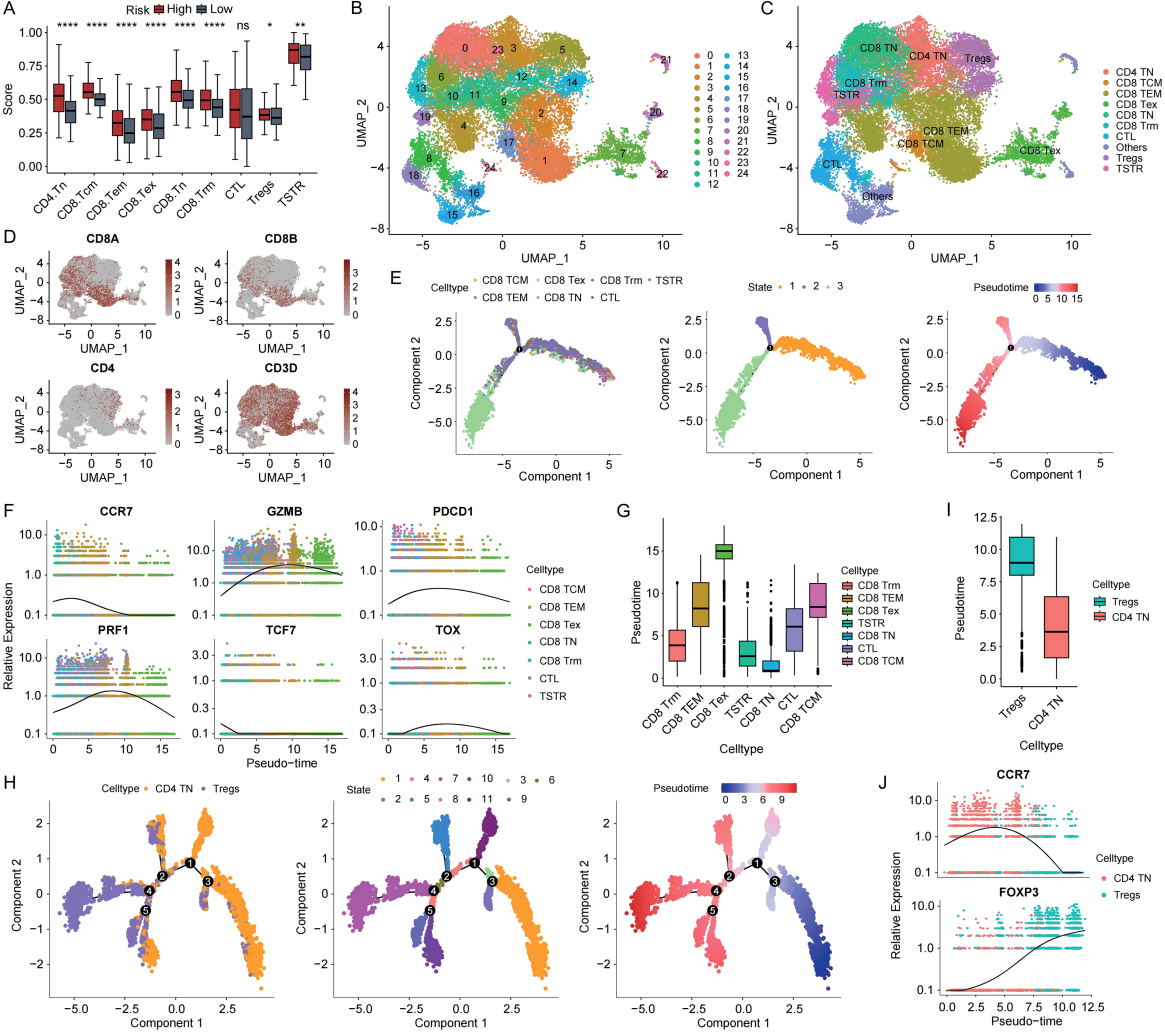
Characterization of T cell heterogeneity and differentiation dynamics in gastric cancer. **(A)** Boxplot illustrating differential T lymphocyte infiltration stratified by risk status in the TCGA cohort. **(B)** UMAP projection depicting T cell clustering patterns in gastric cancer scRNA-seq data. **(C)** UMAP plot displaying re-annotated T cell subtypes after refinement. **(D)** Expression patterns of representative marker genes across T cell subpopulations. **(E)** Pseudotime trajectory illustrating the differentiation path of CD8^+^ T cell subsets. **(F)** Dynamic expression trends of key marker genes during CD8^+^ T cell differentiation. **(G)** Boxplot of pseudotime distributions among distinct CD8^+^ T cell subsets. **(H)** Pseudotime trajectory of CD4^+^ T cell subset differentiation. **(I)** Pseudotime distribution of various CD4^+^ T cell subtypes visualized by boxplot. **(J)** Expression dynamics of marker genes along the CD4^+^ T cell differentiation continuum. TN: naïve T cells; Tex: exhausted T cells; TSTR: T cell stress response state; TEM: effector memory T cells; CTL: Cytotoxic T Lymphocytes; Tregs: regulatory T cells; TCM: central memory T cells. *p<0.05, **p<0.01, ****p<0,0001.

Based on ssGSEA using T cell markers from a previously published study (16), we observed significantly higher T cell infiltration in high-risk patients from the TCGA cohort (**Figure 7A**). To further characterize T cell dynamics, we re-annotated 27,703 single T cells into CD4+ and CD8+ subsets (**Figures 7B, C**), and confirmed marker expression at the single-cell level (**Figure 7D**). In CD8+ T cells, early-stage markers such as CCR7 and TCF7 were highly expressed at the beginning of the trajectory, while genes associated with effector function and exhaustion like GZMB, PRF1, PDCD1 and TOX showed increasing expression toward the terminal states (**Figures 7E, F**). Pseudotime distributions confirmed a progression from CD8+ TN (naïve) cells to CD8+ Tex (exhausted) cells, with Tex cells occupying more advanced positions along the trajectory (**Figure 7G**). A similar pattern was observed in CD4+ T cells. CCR7 was predominantly expressed in early-stage CD4+ TN cells, while FOXP3, a hallmark of regulatory T cells (Tregs), was enriched in cells at later pseudotime stages (**Figures 7H–J**), reflecting a transition toward immunosuppressive phenotypes. These results highlight the continuous differentiation of tumor-infiltrating T cells in gastric cancer, offering insights into their functional states within the immune microenvironment.

### Genomic alterations and therapeutic vulnerabilities

**Figure 8:**
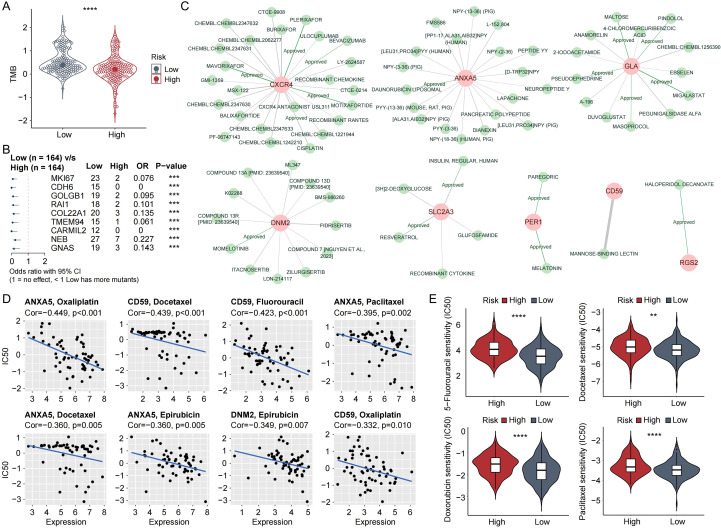
Analysis of tumor mutation burden and drug sensitivity in relation to risk stratification. **(A)** Tumor mutational burden (TMB) disparity across prognostic risk strata. **(B)** Differential mutational landscapes identifying high-impact genes in TCGA-STAD cohorts. **(C)** Predicted drug interactions with the identified signature genes. **(D)** Signature gene expression correlates with pharmacogenomic sensitivity in CellMiner repository. **(E)** Violin plots showing sensitivity differences to four representative drugs between high- and low-risk groups. ns: not significant, **p<0.01, ***p<0.001, ****p<00001.

Analysis of TMB demonstrated that the low-risk cohort exhibited a significantly elevated total mutational burden. (**Figure 8A**, **Supplementary Figure 10**). The top nine significantly mutated genes, including MKI67, CDH6 and GOLGB1 were more frequently mutated in low-risk patients (**Figure 8B**). Potential drug-gene interactions involving the identified signature genes were explored using DGIdb (**Figure 8C**). Furthermore, analysis using the CellMiner database revealed several negative correlations between key genes and drug sensitivities. ANXA5 and CD59 negatively correlated with Oxaliplatin and Docetaxel; CD59 also correlated with Fluorouracil, and DNM2 with Epirubicin (**Figure 8D**). Consistently, drug-response prediction using *pRRophetic* estimated lower IC50 values for 5-Fluorouracil, Docetaxel, Doxorubicin, and Paclitaxel in the low-risk group (**Figure 8E**), indicating a potential increased chemosensitivity in this cohort. These computational inferences derive from established *in vitro* pharmacogenomic datasets and thus represent hypotheses about differential drug response that warrant follow-up experimental and clinical validation to determine their translational relevance.

## Discussion

This study delineates the pivotal role of polyamine metabolism in shaping the TME and influencing patient prognosis in GC through an integrated analysis. Combining single-cell and bulk transcriptomic data, we reveal that molecular features associated with polyamine metabolism promote GC progression by orchestrating immune evasion, stromal activation, and therapy resistance.

Within the 13-gene prognostic signature, several genes were associated with tumor immunity and metabolic remodeling in GC. ANXA5 and CXCR4 have been implicated in promoting immune suppression and metastasis in GC (17, 18), while CD59 mediates complement-based immune evasion (19). SLC2A3, a glucose transporter, enhances glycolytic flux and provides metabolic intermediates that may support polyamine biosynthesis (20). ZFP36 regulates mRNA stability of inflammatory mediators (21), and RGS2 and PER1 participate in cellular stress and circadian control, which may indirectly modulate immune and metabolic homeostasis (22, 23). CDK5RAP3 and ARGLU1 function in transcriptional and cell cycle regulation (24, 25), whereas TAP1 and TCIRG1 contribute to antigen presentation and lysosomal acidification, potentially affecting the tumor immune microenvironment (26, 27). GLA and DNM2 are involved in membrane remodeling and metabolic adaptation (28, 29). However, since our conclusions are primarily derived from computational inference, the proposed immunological and metabolic roles of these genes should be interpreted with caution. Future studies integrating *in vitro* functional assays, metabolic flux tracing, and immune co-culture experiments are warranted to validate their mechanistic links to polyamine metabolism and immune regulation in gastric cancer.

Functional enrichment analysis revealed significant activation of stromal remodeling-related pathways like TGF-β signaling, focal adhesion and ECM-receptor interaction in high-risk patients, consistent with higher ESTIMATE stromal scores, suggesting enhanced stromal activity may impede immune cell infiltration (16, 30).In contrast, low-risk patients showed enrichment in nucleic acid repair and RNA degradation pathways, accompanied by higher TMB, which may reflect increased neoantigen generation and a more immune-sensitive phenotype (31, 32). Notably, patients with GS tumors demonstrated higher risk scores, suggesting that a high-risk score may capture a stromal- and tumor-driven phenotype with lower genomic instability. In these patients, higher proportions of cancer and mast cells were observed, potentially linking genomic stability with tumor composition and risk stratification.

Single-cell communication analysis revealed enhanced outgoing signals from fibroblasts and macrophages in high-risk tumors, enriched in SPP1, TNF, and ANNEXIN pathways, which are computationally predicted to promote immunosuppressive macrophage polarization, T cell dysfunction, angiogenesis, and stromal remodeling (33–38). Low-risk tumors showed tumor cell-dominated signals like PTN and ncWNT pathways mainly associated with growth regulation rather than immune suppression (39, 40).

Single-cell pseudotime analysis suggested predicted differentiation trajectories of both CD8^+^ and CD4^+^ T cells transitioning from naïve to exhausted states (41). Notably, high-risk tumors exhibited concurrent high T cell infiltration and T cell exhaustion, as evidenced by increased abundance of CD8^+^ effector T cells alongside upregulated exhaustion markers (PDCD1, TOX) and enrichment of regulatory T cells (Tregs). These observations represent computational predictions rather than confirmed causal events. They indicate an immunologically “hot” yet functionally impaired microenvironment, where T cells may be recruited or expanded in response to tumor antigens but are likely constrained by chronic stimulation, immunosuppressive cues from stromal and myeloid cells, and metabolic factors associated with polyamine metabolism (42). The coexistence of immune activation and suppression likely contributes to the poor prognosis observed in high-risk patients. Enriched stromal interactions, particularly SPP1 and TNF signaling from fibroblasts and macrophages, may further exacerbate T cell dysfunction and promote Treg-mediated immunosuppression. This duality underscores the complexity of the TME in high-risk GC and suggests that polyamine metabolism orchestrates both immune recruitment and exhaustion, with implications for immunotherapeutic strategies.

Immune profiling indicated that high-risk tumors exhibited increased infiltration of CD8^+^ T cells, Tregs, DCs, and macrophages, alongside elevated immune checkpoint expression and higher TIDE scores, reflecting functional suppression of immune responses (43). Correspondingly, low-risk patients demonstrated superior predicted immunotherapy sensitivity based on IPS and IMvigor210 cohort analysis, which may be partially explained by higher TMB and lower immunosuppressive activity. Drug sensitivity analyses further suggested that low-risk patients might respond better to chemotherapy, although these findings are computational inferences and require clinical validation.

Several limitations warrant acknowledgment. First, the 13-gene signatures and their functional links to polyamine metabolism are largely derived from computational analyses and literature inference; no direct *in vitro* or *in vivo* functional experiments were performed. Second, single-cell datasets were limited to publicly available cohorts and may not capture population-wide heterogeneity. Third, drug sensitivity and immunotherapy response predictions are based on computational models and require experimental and clinical confirmation. Notably, the prognostic model shows moderate performance (AUC 0.64–0.67), indicating it may be more suitable for risk stratification and biological insight than for direct clinical decision-making. These findings are based on computational analyses of public datasets, which may not capture full population heterogeneity and warrant prospective experimental validation.

In summary, this study presents a multi-omics framework linking polyamine metabolism with GC progression, immune modulation, and stromal activation. The 13-gene prognostic model stratifies patients by risk and highlights candidate biomarkers for further exploration in experimental and therapeutic studies.

## Conclusion

Polyamine metabolism appears to influence gastric cancer progression by modulating the tumor microenvironment, including stromal activation and immune suppression. We developed and validated a 13-gene prognostic model that effectively stratifies patients into high- and low-risk groups with distinct immune profiles, genomic features, and predicted therapy sensitivities. These findings provide a foundation for future functional studies and may inform personalized strategies targeting polyamine metabolism in gastric cancer.

## Data Availability

The original contributions presented in the study are included in the article/**Supplementary Material**. Further inquiries can be directed to the corresponding author.

## References

[1] Chen Y Jia K Xie Y Yuan J Liu D Jiang L . The current landscape of gastric cancer and gastroesophageal junction cancer diagnosis and treatment in China: a comprehensive nationwide cohort analysis. J Hematol Oncol. (2025) 18:42. doi: 10.1186/s13045-025-01698-y, PMID: 40234884 PMC12001465

[2] Ajani JA D’Amico TA Bentrem DJ Chao J Cooke D Corvera C . Gastric cancer, version 2.2022, NCCN clinical practice guidelines in oncology. J Natl Compr Cancer Netw: JNCCN. (2022) 20:167–92. doi: 10.6004/jnccn.2022.0008, PMID: 35130500

[3] Yang WJ Zhao HP Yu Y Wang JH Guo L Liu JY . Updates on global epidemiology, risk and prognostic factors of gastric cancer. World J Gastroenterol. (2023) 29:2452–68. doi: 10.3748/wjg.v29.i16.2452, PMID: 37179585 PMC10167900

[4] Assumpção P Araújo T Khayat A Ishak G Santos S Barra W . Hereditary gastric cancer: Three rules to reduce missed diagnoses. World J Gastroenterol. (2020) 26:1382–93. doi: 10.3748/wjg.v26.i13.1382, PMID: 32308342 PMC7152522

[5] Zhang W . TCGA divides gastric cancer into four molecular subtypes: implications for individualized therapeutics. Chin J Cancer. (2014) 33:469–70. doi: 10.5732/cjc.014.10117, PMID: 25223913 PMC4198748

[6] Yasuda T Wang YA . Gastric cancer immunosuppressive microenvironment heterogeneity: implications for therapy development. Trends Cancer. (2024) 10:627–42. doi: 10.1016/j.trecan.2024.03.008, PMID: 38600020 PMC11292672

[7] Holbert CE Cullen MT Casero RA Jr. Stewart TM . Polyamines in cancer: integrating organismal metabolism and antitumour immunity. Nat Rev Cancer. (2022) 22:467–80. doi: 10.1038/s41568-022-00473-2, PMID: 35477776 PMC9339478

[8] Schibalski RS Shulha AS Tsao BP Palygin O Ilatovskaya DV . The role of polyamine metabolism in cellular function and physiology. Am J Physiol Cell Physiol. (2024) 327:C341–c56. doi: 10.1152/ajpcell.00074.2024, PMID: 38881422 PMC11427016

[9] Sagar NA Tarafdar S Agarwal S Tarafdar A Sharma S . Polyamines: functions, metabolism, and role in human disease management. Med Sci (Basel Switzerland). (2021) 9:44. doi: 10.3390/medsci9020044, PMID: 34207607 PMC8293435

[10] Novita Sari I Setiawan T Seock Kim K Toni Wijaya Y Won Cho K Young Kwon H . Metabolism and function of polyamines in cancer progression. Cancer Lett. (2021) 519:91–104. doi: 10.1016/j.canlet.2021.06.020, PMID: 34186159

[11] Xu L You X Cao Q Huang M Hong LL Chen XL . Polyamine synthesis enzyme AMD1 is closely associated with tumorigenesis and prognosis of human gastric cancers. Carcinogenesis. (2020) 41:214–22. doi: 10.1093/carcin/bgz098, PMID: 31140554

[12] Duan Y Xu Y Dou Y Xu D . Helicobacter pylori and gastric cancer: mechanisms and new perspectives. J Hematol Oncol. (2025) 18:10. doi: 10.1186/s13045-024-01654-2, PMID: 39849657 PMC11756206

[13] Chaturvedi R de Sablet T Coburn LA Gobert AP Wilson KT . Arginine and polyamines in Helicobacter pylori-induced immune dysregulation and gastric carcinogenesis. Amino Acids. (2012) 42:627–40. doi: 10.1007/s00726-011-1038-4, PMID: 21874531 PMC3258477

[14] Sun K Xu R Ma F Yang N Li Y Sun X . scRNA-seq of gastric tumor shows complex intercellular interaction with an alternative T cell exhaustion trajectory. Nat Commun. (2022) 13:4943. doi: 10.1038/s41467-022-32627-z, PMID: 35999201 PMC9399107

[15] Wen F Guan X Qu HX Jiang XJ . Integrated analysis of single-cell and bulk RNA-seq establishes a novel signature for prediction in gastric cancer. World J Gastrointestinal Oncol. (2023) 15:1215–26. doi: 10.4251/wjgo.v15.i7.1215, PMID: 37546563 PMC10401466

[16] Wen Z Wang L Ma H Li L Wan L Shi L . Integrated single-cell transcriptome and T cell receptor profiling reveals defects of T cell exhaustion in pulmonary tuberculosis. J Infect. (2024) 88:106158. doi: 10.1016/j.jinf.2024.106158, PMID: 38642678

[17] Hong Z Wen P Wang K Wei X Xie W Rao S . The macrophage-associated prognostic gene ANXA5 promotes immunotherapy resistance in gastric cancer through angiogenesis. BMC Cancer. (2024) 24:141. doi: 10.1186/s12885-024-11878-7, PMID: 38287304 PMC10823665

[18] Patel B Silwal A Eltokhy MA Gaikwad S Curcic M Patel J . Deciphering CD59: unveiling its role in immune microenvironment and prognostic significance. Cancers. (2024) 16:3699. doi: 10.3390/cancers16213699, PMID: 39518137 PMC11545456

[19] Zhao H Jiang R Zhang C Feng Z Wang X . The regulatory role of cancer stem cell marker gene CXCR4 in the growth and metastasis of gastric cancer. NPJ Precis Oncol. (2023) 7:86. doi: 10.1038/s41698-023-00436-2, PMID: 37679408 PMC10484911

[20] Yao X He Z Qin C Deng X Bai L Li G . SLC2A3 promotes macrophage infiltration by glycolysis reprogramming in gastric cancer. Cancer Cell Int. (2020) 20:503. doi: 10.1186/s12935-020-01599-9, PMID: 33061855 PMC7552479

[21] Angiolilli C Leijten EFA Bekker CPJ Eeftink E Giovannone B Nordkamp MO . ZFP36 family members regulate the proinflammatory features of psoriatic dermal fibroblasts. J Invest Dermatol. (2022) 142:402–13. doi: 10.1016/j.jid.2021.06.030, PMID: 34333017

[22] Yang S Sun B Li W Yang H Li N Zhang X . Fatty acid metabolism is related to the immune microenvironment changes of gastric cancer and RGS2 is a new tumor biomarker. Front Immunol. (2022) 13:1065927. doi: 10.3389/fimmu.2022.1065927, PMID: 36591293 PMC9797045

[23] Wang J Huang Q Hu X Zhang S Jiang Y Yao G . Disrupting circadian rhythm via the PER1-HK2 axis reverses trastuzumab resistance in gastric cancer. Cancer Res. (2022) 82:1503–17. doi: 10.1158/0008-5472.CAN-21-1820, PMID: 35255118 PMC9662874

[24] Wang JB Gao YX Ye YH Lin TX Li P Lin JX . CDK5RAP3 acts as a tumour suppressor in gastric cancer through the infiltration and polarization of tumour-associated macrophages. Cancer Gene Ther. (2023) 30:22–37. doi: 10.1038/s41417-022-00515-9, PMID: 35999359 PMC9842504

[25] Li F Li J Yu J Pan T Yu B Sang Q . Identification of ARGLU1 as a potential therapeutic target for gastric cancer based on genome-wide functional screening data. EBioMedicine. (2021) 69:103436. doi: 10.1016/j.ebiom.2021.103436, PMID: 34157484 PMC8220577

[26] Tu Z Li K Ji Q Huang Y Lv S Li J . Pan-cancer analysis: predictive role of TAP1 in cancer prognosis and response to immunotherapy. BMC Cancer. (2023) 23:133. doi: 10.1186/s12885-022-10491-w, PMID: 36759763 PMC9912572

[27] Xu C Jia B Yang Z Han Z Wang Z Liu W . Integrative analysis identifies TCIRG1 as a potential prognostic and immunotherapy-relevant biomarker associated with Malignant cell migration in clear cell renal cell carcinoma. Cancers. (2022) 14:4583. doi: 10.3390/cancers14194583, PMID: 36230507 PMC9558535

[28] Zhang Y Li J Yin X . High-expression of Galactosidase alpha is correlated with poor prognosis and immune infiltration in low-grade glioma. J Cancer. (2023) 14:646–56. doi: 10.7150/jca.81975, PMID: 37057282 PMC10088540

[29] Trochet D Bitoun M . A review of Dynamin 2 involvement in cancers highlights a promising therapeutic target. J Exp Clin Cancer Res: CR. (2021) 40:238. doi: 10.1186/s13046-021-02045-y, PMID: 34294140 PMC8296698

[30] Mai Z Lin Y Lin P Zhao X Cui L . Modulating extracellular matrix stiffness: a strategic approach to boost cancer immunotherapy. Cell Death Dis. (2024) 15:307. doi: 10.1038/s41419-024-06697-4, PMID: 38693104 PMC11063215

[31] Park S Lee H Lee B Lee SH Sun JM Park WY . DNA damage response and repair pathway alteration and its association with tumor mutation burden and platinum-based chemotherapy in SCLC. J Thorac Oncol. (2019) 14:1640–50. doi: 10.1016/j.jtho.2019.05.014, PMID: 31125737

[32] Budczies J Kazdal D Menzel M Beck S Kluck K Altbürger C . Tumour mutational burden: clinical utility, challenges and emerging improvements. Nat Rev Clin Oncol. (2024) 21:725–42. doi: 10.1038/s41571-024-00932-9, PMID: 39192001

[33] Huang Z Li Y Liu Q Chen X Lin W Wu W . SPP1-mediated M2 macrophage polarization shapes the tumor microenvironment and enhances prognosis and immunotherapy guidance in nasopharyngeal carcinoma. Int Immunopharmacol. (2025) 147:113944. doi: 10.1016/j.intimp.2024.113944, PMID: 39742726

[34] Nair R Somasundaram V Kuriakose A Krishn SR Raben D Salazar R . Deciphering T-cell exhaustion in the tumor microenvironment: paving the way for innovative solid tumor therapies. Front Immunol. (2025) 16:1548234. doi: 10.3389/fimmu.2025.1548234, PMID: 40236693 PMC11996672

[35] Araújo TG Mota STS Ferreira HSV Ribeiro MA Goulart LR Vecchi L . Annexin A1 as a regulator of immune response in cancer. Cells. (2021) 10:2245. doi: 10.3390/cells10092245, PMID: 34571894 PMC8464935

[36] Du Y Lin Y Gan L Wang S Chen S Li C . Potential crosstalk between SPP1 + TAMs and CD8 + exhausted T cells promotes an immunosuppressive environment in gastric metastatic cancer. J Trans Med. (2024) 22:158. doi: 10.1186/s12967-023-04688-1, PMID: 38365757 PMC10870525

[37] Jiang W He Y He W Wu G Zhou X Sheng Q . Exhausted CD8+T cells in the tumor immune microenvironment: new pathways to therapy. Front Immunol. (2020) 11:622509. doi: 10.3389/fimmu.2020.622509, PMID: 33633741 PMC7902023

[38] Xiao D Zeng T Zhu W Yu ZZ Huang W Yi H . ANXA1 promotes tumor immune evasion by binding PARP1 and upregulating stat3-induced expression of PD-L1 in multiple cancers. Cancer Immunol Res. (2023) 11:1367–83. doi: 10.1158/2326-6066.CIR-22-0896, PMID: 37566399

[39] He J Tian F Li J Zhang Y Chu Z . The lncrna HMMR-AS1 promotes the Malignant progression of ovarian cancer cells by regulating the miR-627-3p/PTN axis. J Ovarian Res. (2025) 18:119. doi: 10.1186/s13048-025-01691-6, PMID: 40462168 PMC12131470

[40] Haseeb M Pirzada RH Ain QU Choi S . Wnt signaling in the regulation of immune cell and cancer therapeutics. Cells. (2019) 8:1380. doi: 10.3390/cells8111380, PMID: 31684152 PMC6912555

[41] Sun L Su Y Jiao A Wang X Zhang B . T cells in health and disease. Signal Transduction Targeted Ther. (2023) 8:235. doi: 10.1038/s41392-023-01471-y, PMID: 37332039 PMC10277291

[42] Zhu Y Zhou Z Du X Lin X Liang ZM Chen S . Cancer cell-derived arginine fuels polyamine biosynthesis in tumor-associated macrophages to promote immune evasion. Cancer Cell. (2025) 43:1045–60.e7. doi: 10.1016/j.ccell.2025.03.015, PMID: 40185095

[43] Jiang P Gu S Pan D Fu J Sahu A Hu X . Signatures of T cell dysfunction and exclusion predict cancer immunotherapy response. Nat Med. (2018) 24:1550–8. doi: 10.1038/s41591-018-0136-1, PMID: 30127393 PMC6487502

