# Supplementary material for: Integrated analysis of single-cell and bulk transcriptomes reveals the prognostic value of polyamine metabolism biomarkers and immune microenvironment features in gastric cancer
Source: Front Immunol. 2026 Jan 30;16:1658975. doi: 10.3389/fimmu.2025.1658975 (PMC12901417; doi:10.3389/fimmu.2025.1658975)
Supplement: Supplementary file 1 [file DataSheet1.docx]

**Supplementary data**


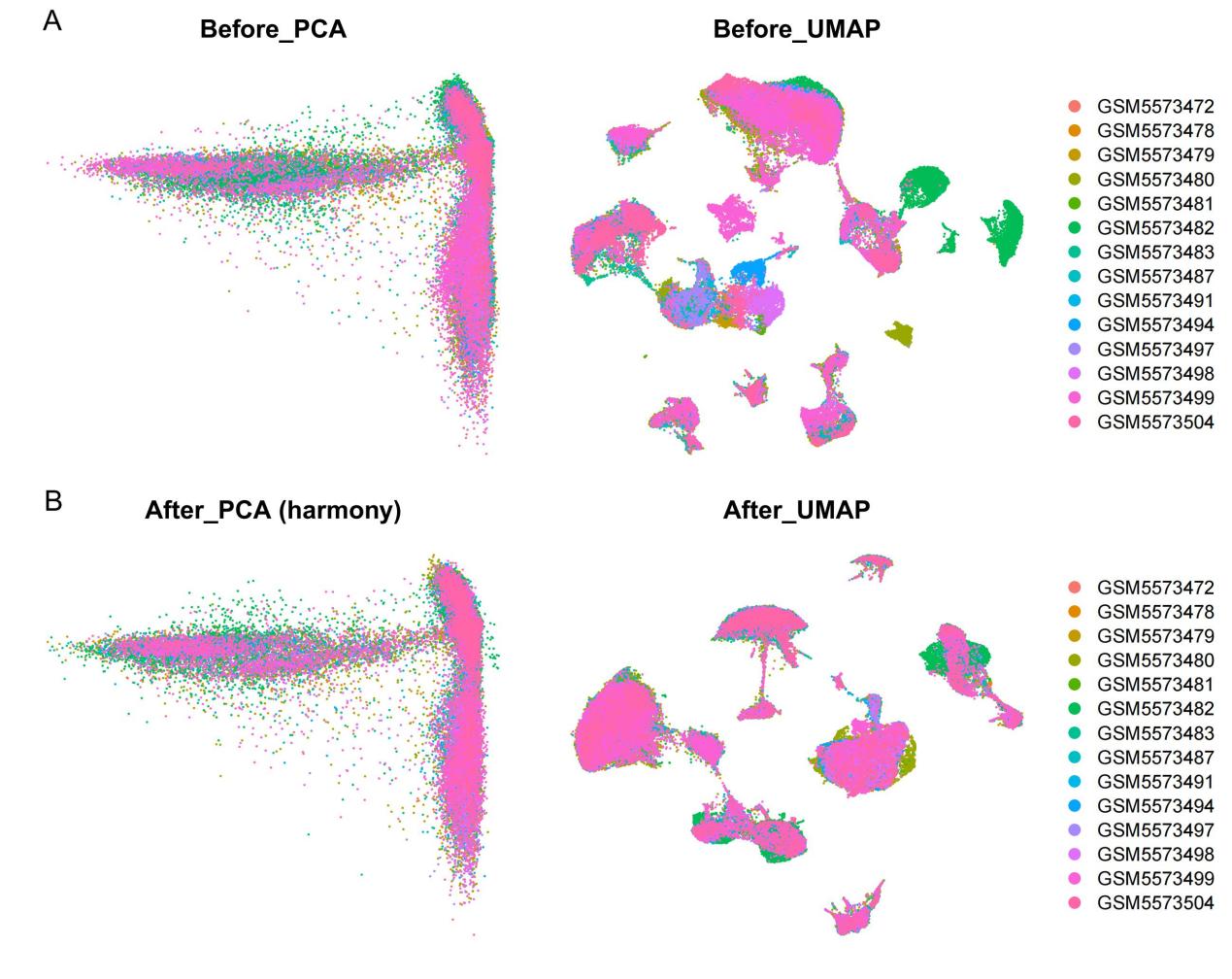


**Supplementary figure 1: Comparison of dimensionality reduction results for single-cell data before and after Harmony batch correction.** (A) Visualization results of PCA dimensionality reduction and UMAP without and (B) after Harmony batch correction.

PCA: Principal Component Analysis; UMAP: Principal Component Analysis


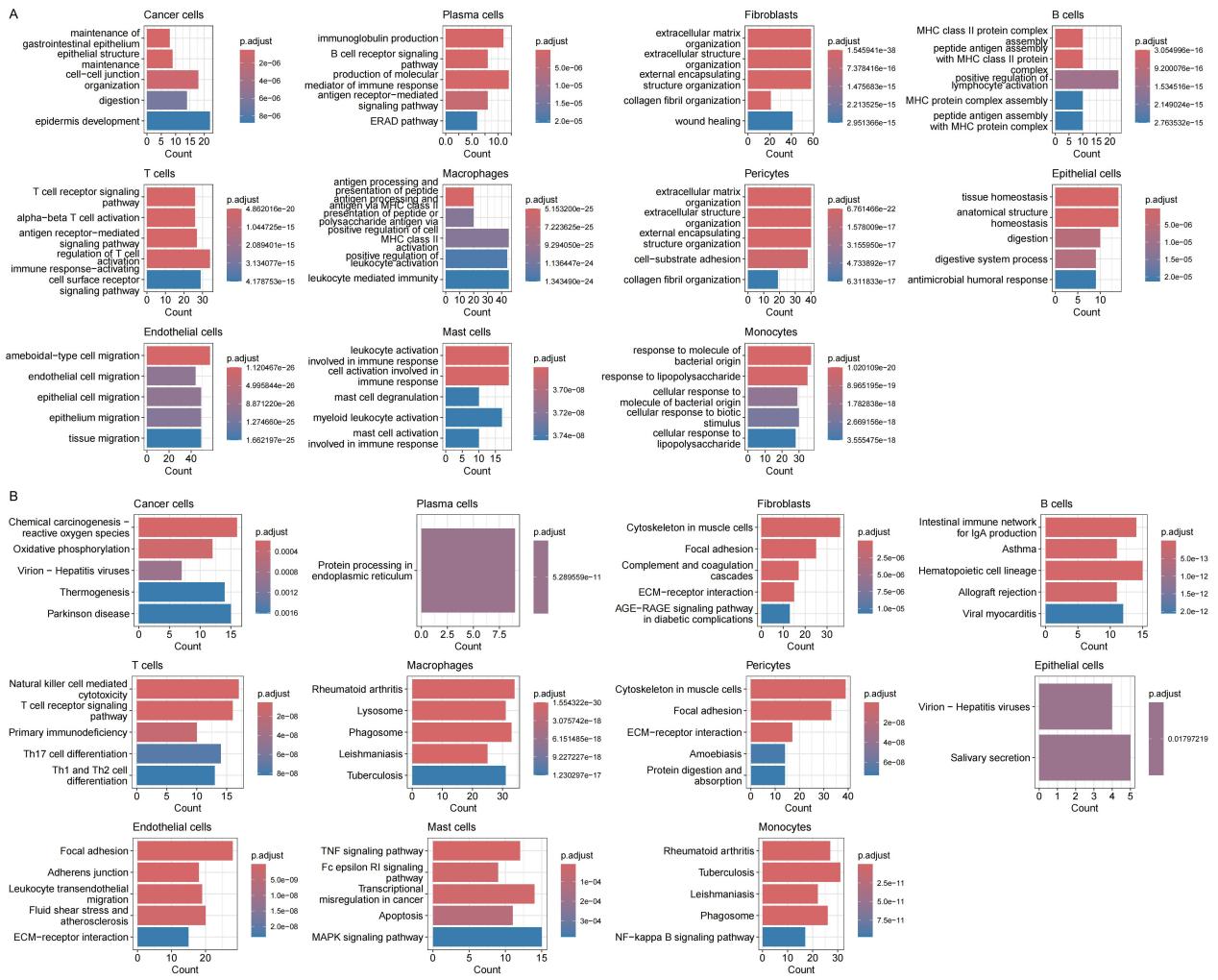


**Supplementary figure 2: Functional enrichment results of differentially expressed genes per cell cluster.** (A) Results of GO and (B) KEGG enrichment analysis for differentially expressed genes in each cell cluster.

GO: Gene Ontology; KEGG: Kyoto Encyclopedia of Genes and Genomes


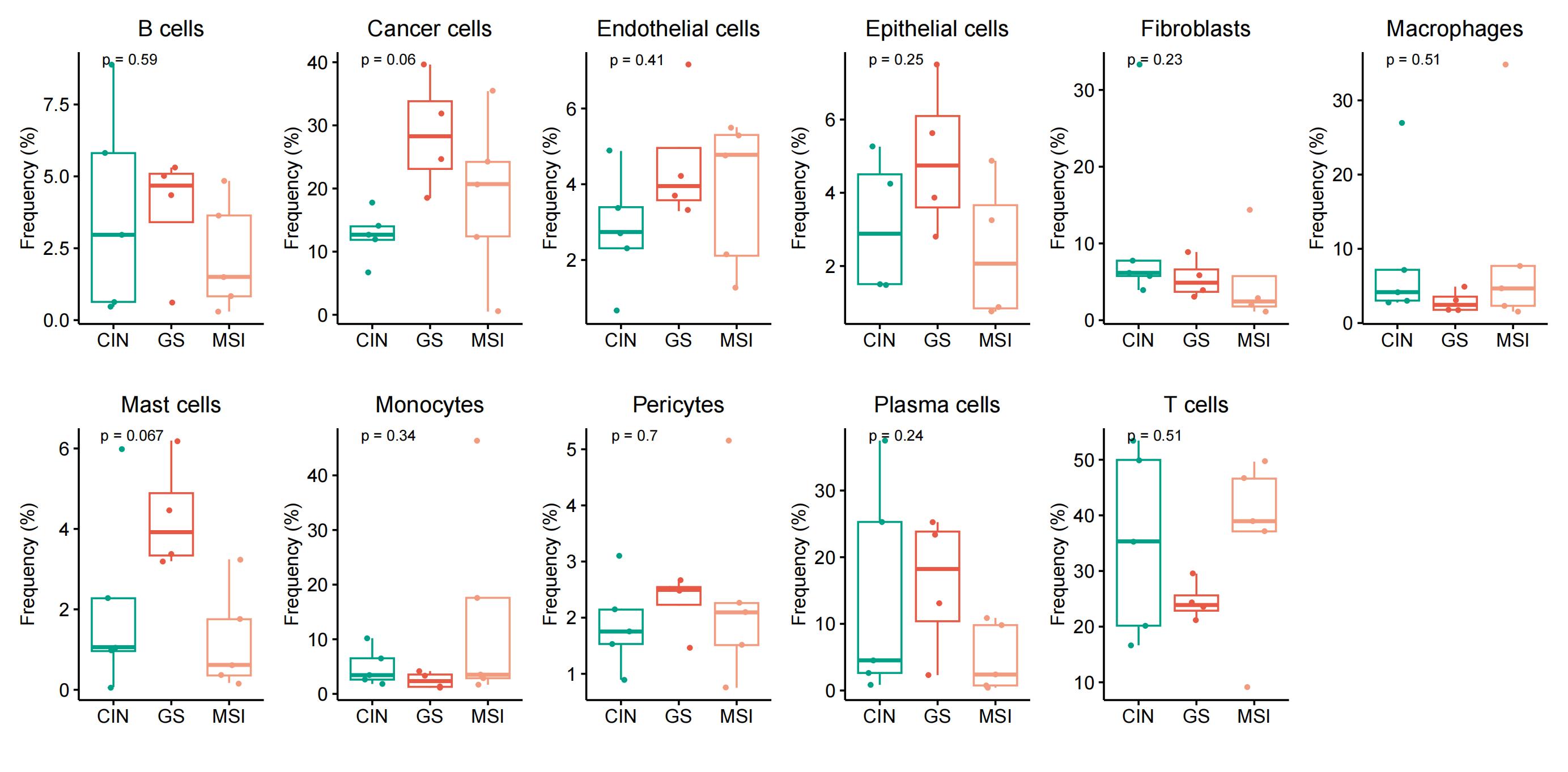


**Supplementary figure 3: Proportions of infiltrating cell types across gastric cancer subtypes at single-cell resolution.**

CIN: Chromosomal Instability; GS: Genomically Stable; MSI: Microsatellite Instability


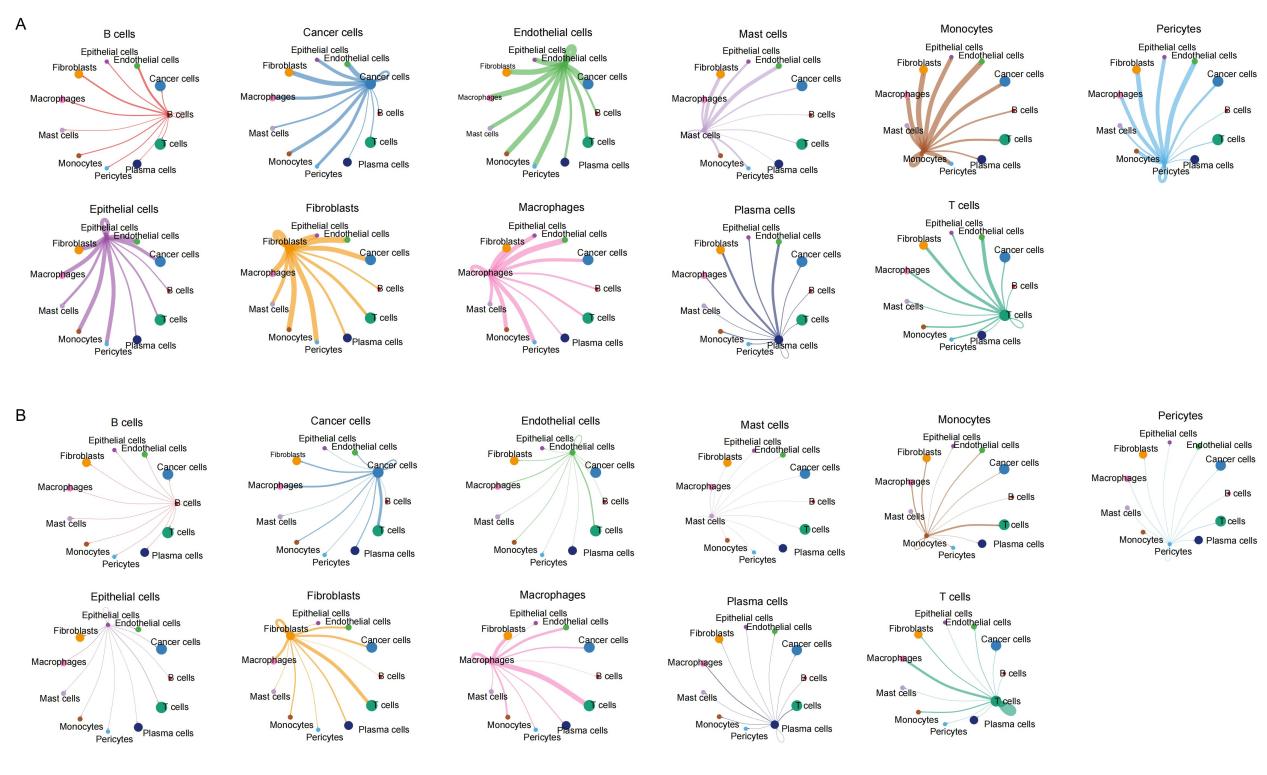


**Supplementary figure 4: Interaction profiles of each cell type with other cell types in high-risk-associated cell populations.** (A) The counts and (B) strength of interactions between major cell types within high-risk-associated cell populations.


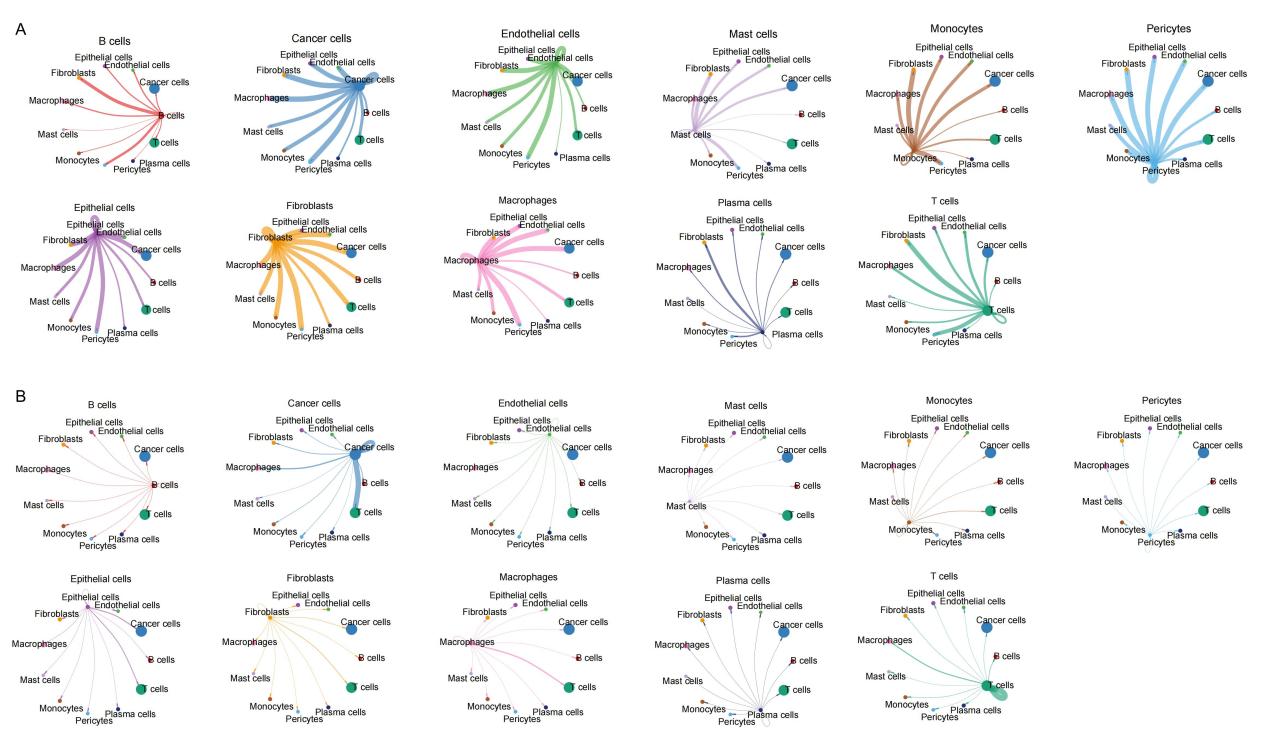


**Supplementary figure 5: Interaction profiles of each cell type with other cell types in low-risk-associated cell populations.** (A) The counts and (B) strength of interactions between major cell types within low-risk-associated cell populations.


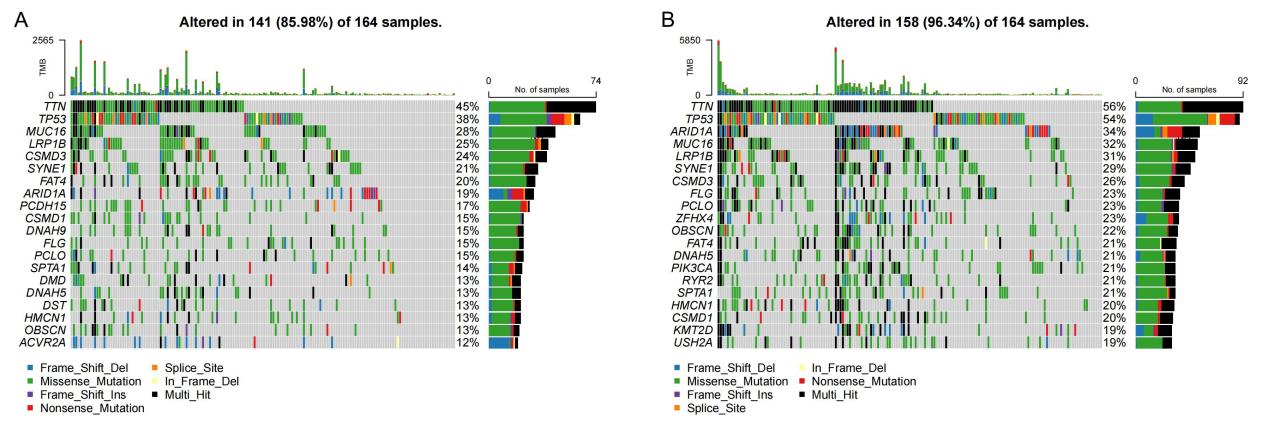


**Supplementary figure 6: Waterfall plot depicting tumor mutation burden (TMB).** (A) TMB of the top 20 genes in high- and (B) low-risk groups.
